# Supplementary material for: Differences in Vegetative, Productive, and Physiological Behaviors in Actinidia chinensis Plants, cv. Gold 3, as A Function of Cane Type
Source: Plants (Basel). 2025 Jul 16;14(14):2199. doi: 10.3390/plants14142199 (PMC12299610; doi:10.3390/plants14142199)
Supplement: Supplementary file 1 [file plants-14-02199-s001.zip › plants-3717097-supplementary.pdf]

Figure S1 - Thermo-pluviometric regime of the 2022–2023 biennium of the area of interest.

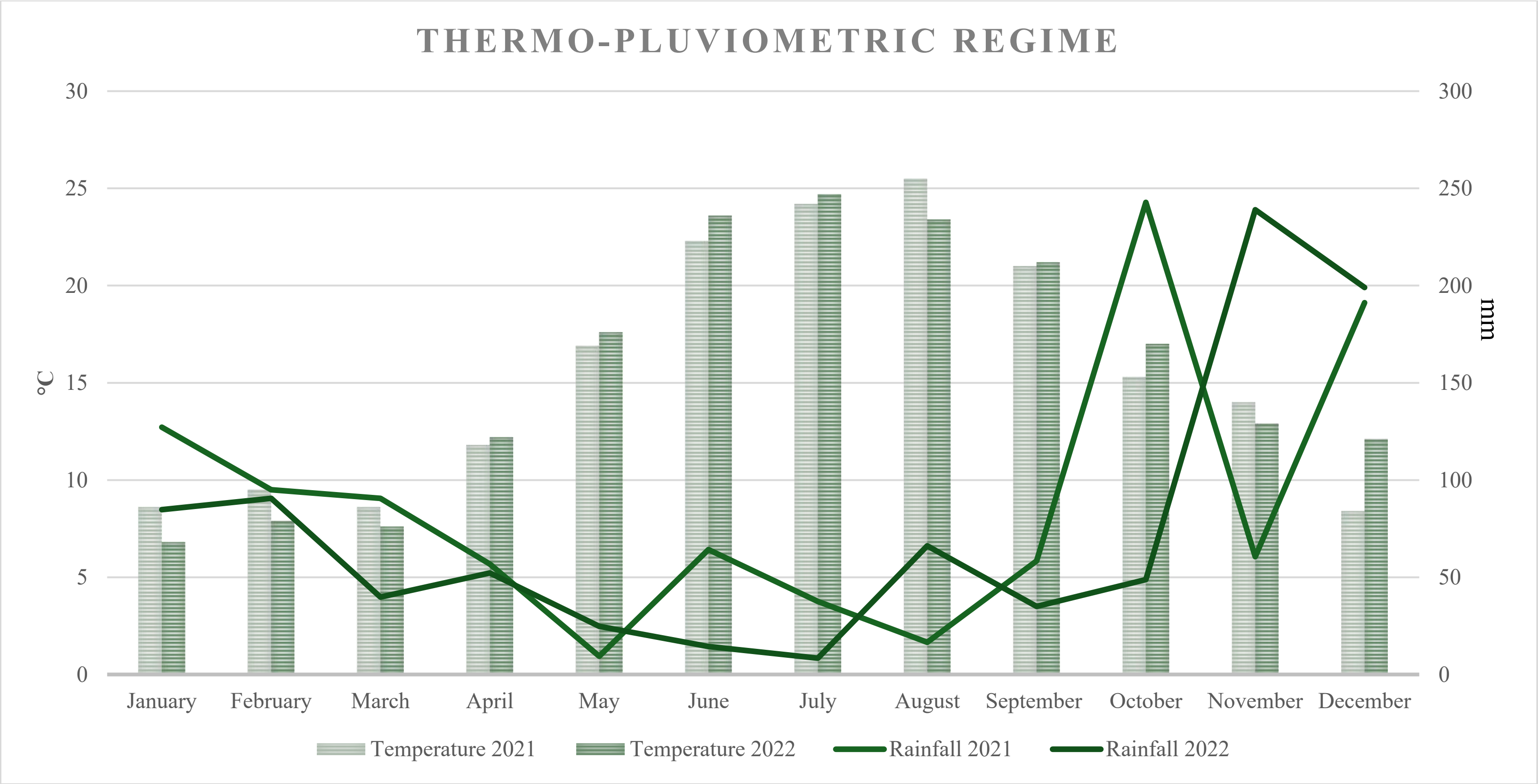

Table S1. ANOVA results for the main ripening indices (TSS, Total soluble solids; Fir, Firmness; TA, Tritratable Acidity) dependent variables. Degrees of freedom (df), F-value, and significance level (p) related to the independent variables [names of the independent variables] are reported.

	The dependent variable								
	TSS			Firmness			TA		
	GL	F	Sign	GL	F	Sign	GL	F	Sign
Model	43	17.668	0.000	32	6.741	0.000	36	426.326	0.000
Intercept	1	11623.915	0.000	1	20056.617	0.000	1	6401.600	0.000
Epoch	3	219.592	0.000	2	38.376	0.000	2	7316.173	0.000
treatment	1	4.654	0.032	1	1.294	0.256	1	3.064	0.081
yeat	1	6.412	0.012	1	4.481	0.035	1	4.061	0.045
Block	2	0.500	0.607	2	0.077	0.926	2	0.280	0.756
Epoch * treatment	3	1.784	0.152	2	3.141	0.045	2	3.064	0.049
Epoch * year	3	1.884	0.134	2	5.908	0.003	2	4.061	0.019
Epoch * Block	5	0.734	0.599	3	0.436	0.727	4	0.280	0.891
treatment * Year	1	0.031	0.860	1	7.211	0.008	1	1.068	0.303
treatment * Block	2	4.548	0.012	2	0.459	0.633	2	1.673	0.190
Year * Block	2	2.008	0.137	2	3.740	0.025	2	1.647	0.195
Epoch * treatment* year	3	0.014	0.998	2	6.666	0.002	2	1.068	0.345
Epoch * treatment * Block	5	4.648	0.000	3	0.043	0.988	4	1.673	0.157
Epoch * year * Block	5	0.727	0.604	3	0.723	0.539	4	1.647	0.164
treatment* year* Block	2	4.543	0.012	2	0.053	0.948	2	2.309	0.102
Epoch * treatment* year * Block	5	3.286	0.007	3	1.262	0.288	4	2.309	0.059
Error	196			231			215		
Total	240			264			252		
Correct Total	239			263			251		

Table S2. ANOVA results for the colorimetric variables (dependent variables). Degrees of freedom (df), F-value, and significance level (p) related to the independent variables [names of the independent variables] are reported.

		L		a		b		Chroma		Hue	
	GL	F	Sign.								
Model	48	15.036	0	70.896	0.000	21.760	0.000	84.176	0.000	78.220	0.000
Intercept	1	141423.6	0	2414.008	0.000	21542.600	0.000	17359.071	0.000	950675.562	0.000
Epoch	4	144.518	0	798.585	0.000	231.735	0.000	930.518	0.000	876.154	0.000
treatment	1	3.893	0.05	2.580	0.110	0.510	0.476	3.006	0.084	1.226	0.269
yeat	1	0.006	0.938	12.051	0.001	5.528	0.020	49.270	0.000	11.161	0.001
Block	2	2.64	0.073	.079	0.924	1.311	0.272	36.124	0.000	0.432	0.650
Epoch * treatment	3	1.57	0.197	5.409	0.001	3.504	0.016	57.199	0.000	5.483	0.001
Epoch * year	3	3.784	0.011	3.408	0.018	2.361	0.072	35.592	0.000	6.968	0.000
Epoch * Block	7	1.961	0.061	0.414	0.893	0.727	0.649	8.924	0.000	0.580	0.772
treatment * Year	1	2.326	0.129	4.458	0.036	5.924	0.016	50.926	0.000	4.224	0.041
treatment * Block	2	2.636	0.074	1.755	0.175	0.041	0.960	25.510	0.000	1.275	0.281
Year * Block	2	1.476	0.23	1.666	0.191	0.456	0.634	1.782	0.170	1.956	0.144
Epoch * treatment* year	3	4.634	0.004	1.579	0.195	3.897	0.010	45.863	0.000	0.098	0.961
Epoch * treatment * Block	6	4.915	0	1.405	0.213	5.250	0.000	14.522	0.000	0.956	0.456
Epoch * year * Block	6	3.307	0.004	2.002	0.066	0.915	0.484	15.328	0.000	2.172	0.046
treatment* year* Block	2	2.056	0.13	2.232	0.109	0.490	0.613	2.010	0.136	1.393	0.250
Epoch * treatment* year * Block	5	1.591	0.163	1.021	0.406	2.569	0.028	18.183	0.000	1.470	0.200
Error	243										
Total	292										
Correct Total	292										
